# Validation of the Chinese Social Constraints Scale Among Malaysian Chinese Postpartum Mothers

**DOI:** 10.21315/mjms-09-2025-617

**Published:** 2026-02-28

**Authors:** Su Rou Low, Suzanna Awang Bono, Zaireeni Azmi, Engku Husna Engku Ismail

**Affiliations:** 1 School of Social Sciences, Universiti Sains Malaysia, Gelugor, Pulau Pinang, Malaysia; 2 Centre for Research on Women and Gender (KANITA), School of Social Sciences, Universiti Sains Malaysia, Gelugor, Pulau Pinang, Malaysia; 3 Department of Obstetrics and Gynaecology, School of Medical Sciences, Universiti Sains Malaysia, Kubang Kerian, Kelantan, Malaysia

**Keywords:** cross-cultural adaption, discriminant analysis, help-seeking behaviour, maternal welfare, psychological adjustment, social environment

## Abstract

**Background:**

In Chinese and collectivist cultures, discussing personal feelings is often considered inappropriate or discouraged, even for postpartum mothers. As a self-reported measure of social responses that inhibit the expression of stressful thoughts, feelings, and experiences, the Social Constraints Scale has been widely validated among cancer and bereaved populations, leaving postpartum mothers unattended.

**Methods:**

This study aimed to assess the psychometric properties of the Chinese version of the Social Constraints Scale (C-SCS) among Chinese postpartum women in Malaysia. A cross-sectional quantitative survey was conducted among 130 Malaysian women in their first postpartum year.

**Results:**

Exploratory factor analysis revealed three underlying factors in the C-SCS, explaining 61.16% of the total variance. Confirmatory factor analysis based on a three-factor structure showed the best fit (*P* < 0.001) after omitting item 3 due to low factor loading. The C-SCS demonstrated excellent reliability with a Cronbach’s alpha value of 0.90 and McDonald’s Omega of 0.90. The three-factor model of the C-SCS exhibited different factor loadings compared with the Greek Social Constraints Scale, suggesting that these differences stem from postpartum and sociocultural contexts.

**Conclusion:**

The C-SCS displayed excellent psychometric properties, making it a reliable and valid measure for screening social constraints and identifying help-seeking barriers among postpartum women in Malaysia.

## Introduction

According to Lepore and Revenson (1), social constraints refer to the objectively and subjectively perceived social conditions that lead individuals to refrain from or modify their disclosure of stress-related emotions and concerns. The concept of social constraints has been described in the social cognitive processing model of adjustment to cancer, suggesting that the social environment influences how individuals cognitively adapt to stressors such as cancer (2). In particular, supportive behaviours are beneficial for coping, whereas unsupportive or constraining behaviours from one’s social network can hinder cognitive processes and lead to greater psychological distress (3).

Lepore et al. (4) first developed a 10- item scale to measure the concept of social constraints, with five items repeated twice for the significant one and others in their social network. The scale was administered among bereaved mothers who lost their infants, and it was found that social constraints moderated the relationship between intrusive thoughts and depressive symptoms, with a higher level of social constraints intensifying the association, and vice versa. Later, Lepore and Ituarte (5) adapted the Social Constraints Scale (SCS) into a 15-item version for trauma survivors, specifically cancer patients, where social constraints were found to act more strongly than social support on the relationship between optimistic and emotional adjustment. Furthermore, social constraints were associated with several mental health indices, including poorer psychological adjustment (6), greater posttraumatic symptoms (7), increased depressive symptoms (8), and greater fear of recurrence (9).

Previous studies have shown that SCS-15 is reliable across diverse backgrounds, including bereaved mothers (4), patients with cancer, and their spouses (5, 10). To date, the SCS has been translated into several languages, including Chinese (11), Turkish (12), Icelandic (13), and Greek (14), and it has demonstrated sound psychometric properties. Although most studies have retained the single-factor model (11–13), a study conducted by Koutrouli et al. (14) on 202 women with cancer proposed a three-factor model of the Greek SCS, showing satisfactory reliability for the unsupportive behaviours, avoidant behaviours, and suggestions for distraction and pretence subscales.

Although SCS-15 was widely validated, studies have primarily focused on cancer diagnosis and bereaved experiences, with no validation of SCS-15 conducted on postpartum experience. It has been argued that childbirth is similar to other traumatic experiences. The tremendous physical and psychological changes that occur during childbirth can overwhelm mothers with emotions and leave them in a vulnerable state, especially among mothers pressured by societal expectations of being a “good” mother (15–17). As indicated, Malaysians have reported a prevalence of postpartum depression (PPD) ranging from 5.1% to 48.6% (18–20), with the duration of onset ranging from the first months to two years postpartum, raising public concern about maternal mental health due to its associated detrimental consequences and duration of the pre-onset period. Nevertheless, existing scales primarily assess women’s experiences as mothers or focus on childbirth (21, 22), with no scale specifically evaluating the experience of being belittled and ignored by postdelivery women as individuals.

Additionally, mothers are sensitive to simple words or actions during their postpartum period (23). The arrival of a newborn is often accompanied by an expectation that motherhood should be joyful and exciting, leading to little expressions of concern for mothers’ mental health from their families, friends, and partners, leaving mothers feeling isolated and struggling to adapt to their new role (24). In Malaysia’s collectivist culture, the stigma surrounding mental illness and expectations of maternal self-sacrifice discourage personal emotional expression and disclosure of PPD symptoms, leading many mothers to suffer in silence (25–27). Fear of judgement, shame, and appearing inadequate not only intensifies symptoms of PPD but also creates social constraints that hinder mothers from seeking support or professional help (28–30). Considering that the social constraints women may face after childbirth greatly depend on their sociocultural context, it is important to adapt the scale and assess its validity when used in new contexts (i.e., among postpartum mothers), given that the scale was initially designed for cancer or bereaved populations.

Furthermore, studies indicate that cultural accommodation occurs when questionnaires are administered without specific cultural context, influencing response styles across different languages, implying the need to translate and validate scales despite being administered in the same country (31). Considering Malaysia’s multicultural society, where diverse ethnicities practice different cultures and speak different languages or dialects, the psychometric properties of the SCS-15 were investigated in a cultural or life event setting different from its original development. This study aims to enhance its theoretical and empirical basis and provide evidence of its stability or variability in other settings, specifically among postpartum women in Malaysia. Therefore, the study adapted and psychometrically assessed the SCS-15 among postpartum women in Malaysia.

## Methods

### Study Design/Participants

This was a cross-sectional study. Purposive and snowball sampling were used to recruit postpartum women. Study eligibility was restricted to Malaysian women aged 18 to 40 years old, literate in Chinese, married within the first 12 months postpartum, who had delivered a healthy baby, and who were not diagnosed with any intellectual disabilities or psychotic disorders. Ethical approval was obtained from the authors’ institution and the Ministry of Health.

A sample size of 75 participants was required for 15 items, based on the subject-to-item ratio of 5:1 required for factor analysis (32). To compensate for a 40% non-response rate in the self-administered survey, an additional 50 participants were included in this study, resulting in a sample size of 125 participants. The additional sample size to compensate for non-response and missing data was calculated based on the formula proposed by Bujang (33) as follows:


75/0.6=125 participants

### Instruments

#### SCS

Lepore and Ituarte (5) developed the SCS-15. The Greek version of the SCS (14) was used to assess the full scale. The SCS is a 15-item scale that measures the frequency with which participants felt socially constrained when interacting with people in their social network during the past month. Participants were asked to rate social constraints from two domains: spouse and family/friends. For each item, participants rated the frequency of socially constraining responses on a four-point frequency scale ranging from 1 (never) to 4 (often). The mean score ranged from 15 to 60, with a higher mean score indicating a higher frequency of social constraints experiences.

To examine the criterion and discriminant validity of the C-SCS, other variables, such as postpartum support and postpartum depression, were measured. The following instruments were used to assess these variables.

#### Postpartum Support Questionnaire (PSQ)

Participants’ emotional support was measured using the 10-item emotional support subscale extracted from the original 34-item PSQ (34). The PSQ is a self-report instrument that measures four types of social support—material, emotional, informational, and comparison support—provided to women after childbirth. Each PSQ item is specific to the support that a woman commonly needs as she adjusts to parenting and the maternal role. Items were rated using a response format of eight options ranging from 0 (not important/no support) to 7 (very important/much support). The scales are summed separately for importance and support. The total score ranges from 0 to 70, with higher scores indicating higher importance or more support expected or received.

#### Edinburgh Postnatal Depression Scale

PPD was measured using the Edinburgh Postnatal Depression Scale (EPDS) as described by Cox et al. (35). This self-administered symptom-based screening scale consisted of 10 items, with three reverse-coded items. A 4-point scale ranging from 0 to 3 was used to assess PPD among postpartum mothers during the past seven days. The total score of the EPDS ranges from 0 to 30 by summing the score for each item, with higher scores indicating increased severity of depressive symptoms.

### Procedure

#### Scale Adaption

Postpartum mothers often experience psychological distress that disrupts their cognitive processes and subsequently leads to psychological maladjustment, which aligns with the concept of the SPC model. Postpartum distress is primarily centred on emotional struggles, whereas the concerns of patients with cancer, for whom the SCS-15 was originally designed, are more focused on their health conditions. Therefore, modifications to the wording were necessary to ensure that the instrument accurately captures the most relevant aspects of postpartum women’s social constraints. In addition, the PPD experience frequently involves feelings of extreme sadness, worthlessness, helplessness, and numbness that disconnect mothers from recognising their emotions (36). It is important to note that although the terms emotions and feelings are often used interchangeably to refer to the same thing, they are somewhat distinct conceptually. Mothers’ feelings are conscious, subjective emotional experiences or reactions to physical or mental sensations (37). Feelings formed from emotions are easier to identify and recognise (38), since they are the expression and reaction that an individual experiences an emotion (39), even though this occurs at a lower level of consciousness. Beyond feelings, emotions often involve bodily reactions that can only be objectively measured via physiological and behavioural responses (39). Therefore, given that the SCS-15 focuses on how others respond to mothers’ attempts to disclose emotions, acknowledging that mothers can only talk about or express feelings that come to consciousness is crucial. Therefore, the term “feelings,” which is more suitable in the postpartum context, was used to replace the terms “illness” and “health” in the SCS-15.

#### Questionnaire Translation and Validity

The translation was performed following Brislin’s guideline (40), which involved forward translation, back-translation, reconciliation, pretest, and final version documentation. First, the items, instructions, and response anchors of the SCS-15 were forward-translated from English to Chinese by a senior lecturer from a Malaysian public university with more than 10 years of experience in translating Chinese and English. Then, a bilingual solicitor back-translated the SCS-15 into English. The forward and back translations were then examined by a bilingual committee comprising all translators and authors. Experts in social psychology validated the items to ensure they adequately measure the concept of social constraints among postpartum women. The committee discussed the translations and settled minor word-choice and grammatical issues using a consensual approach, resulting in a pre-final version of the C-SCS. While the SCS-15 followed Brislin’s guidelines for translation, the other instruments used for criterion and discriminant validity (i.e., PSQ and EPDS) were also translated following the same procedure as listed above. Finally, the pre-final versions of the SCS-15, PSQ, and EPDS were pre-tested in a small sample of Malaysian postpartum mothers (*n* = 30), and their feedback on the scales’ clarity and semantics was discussed and adopted. The pre-tested SCS-15 (M = 29, SD = 8.10, *α* = 0.90), PSQ (M = 113, SD = 69.31, *α* =.99), and EPDS (M = 11, SD = 6.78, *α* =.92) demonstrated satisfactory reliability.

#### Recruitment of Participants

Data were collected using a paper-and-pen questionnaire and an online survey at four public hospitals across Peninsular Malaysia, while the online survey was created using Google Forms and distributed on Facebook, Instagram, Xiaohongshu, and WhatsApp. Participants were approached once they were identified as conversing in Mandarin or sharing Chinese posts on social media. Before their participation, participants were asked about their Chinese proficiency level and whether they were in their first postpartum year. Participants who were Chinese literate, in their first year postpartum, and who agreed to the Chinese informed consent form were included. Participants were informed about their rights to withdraw and the estimated duration of completing the study (i.e., 15 to 20 minutes), with written consent obtained. For the online survey, participants provided consent by clicking the “agree” button. All information was kept confidential unless required by law. At the end of the survey, all participants were debriefed and provided with contact information for psychological services. All participants consented to the publication of the data provided with no personal identifying information attached. A total of 134 postpartum mothers participated in this study.

#### Statistical Analyses

Data analysis was performed using SPSS version 26.0. Data normality was assessed via skewness and kurtosis tests. A total of 130 responses were retained for inferential analysis. Cronbach’s alpha (α) and McDonald’s Omega (ω) coefficients were computed to address internal consistency. To establish the criterion and discriminant validity, Pearson correlation analyses were conducted using the PSQ (34) and the EPDS (35).

Kaiser–Meyer–Olkin (KMO) measure of sampling adequacy and Bartlett’s test of sphericity were employed to assess whether the data were factorable. First, exploratory factor analysis (EFA) using principal component analysis with Promax rotation was conducted to explore the factor structure of the C-SCS. Promax (oblique) rotation was chosen over orthogonal rotation because correlations were assumed among the factors. The number of factors to be extracted was based on the eigenvalues, with only values that are > 1.0 being retained (41). Only items with loadings ≥ 0.40 were retained for further analysis (42, 43).

Confirmatory factor analysis (CFA) was conducted using IBM SPSS 26.0 AMOS to determine whether the data were consistent with the EFA-suggested model. The chi-squared value, comparative fit index, and root mean square error of approximation were used to evaluate the model fit. A large and statistically significant chi-squared value indicated a poor fit (44). A CFI value greater than 0.90 indicates a good fit, whereas an RMSEA value of 0.05 indicates a close model fit, with values below 0.08 considered acceptable (44).

## Results

### Participant’s Characteristics

Of the 134 participants, four were ineligible (i.e., unmarried). Missing data with less than 5% of the total dataset were replaced with the mean value. Only one missing data point was replaced with a mean age of 29.8 years. The demographic characteristics of the participants are presented in Table 1. The participants’ ages ranged from 19 to 40 years old (M = 29.82 ± 4.28 years, median = 29.0 years). All the women were Chinese. The women reported being married from 1 year to 16 years (M = 3.87 ± 3.00 years, median = 3.00). The majority of participants were Buddhist, had obtained a bachelor’s degree, had a monthly household income ranging from RM 4,850 to RM 10,959, were employed, primiparous, living in an extended family, and had undergone vaginal delivery for their latest childbirth.

### Validity and Reliability

The C-SCS displayed adequate internal consistency with Cronbach’s alpha coefficient (*α* = 0.90; 95% CI: 0.87, 0.92) and McDonald’s omega coefficient (*ω*= 0.90; 95% CI: 0.86, 0.92).

### Criterion and Discriminant Validity

A positive correlation between the C-SCS and the EPDS (*r* = 0.40, *P* < 0.001) indicated satisfactory criterion validity. Additionally, none of the correlations between the C-SCS and either PSQ subscales (i.e., importance of support and support received) were statistically significant, suggesting discriminant validity between social constraints and social support.

### EFA

EFA result showed that all initial communalities were ≥ 0.40, indicating that the proportion of variance in each observed variable accounted for by the remaining variables was satisfactory. The KMO value was 0.87 and *X^2^* (130) value of Bartlett’s test was 960.73, *P* < 0.001, suggesting that the C-SCS was suitable for factor analysis. The factor solution derived from this analysis yielded three factors for the scale, explaining 61.16% of the data variance.

As shown in Table 2, all items were single-loading items, with high loadings on their respective factors. The first factor, which accounted for 43.45% of the variance, comprised 10 items of unsupportive behaviours. The second factor, which explained 9.69% of the variance, consisted of three items indicating avoidant behaviours. The third factor, explaining 8.02% of the variance and comprising two items, appeared to reflect suggestions for distraction and pretence.

### CFA

**Figure 1:**
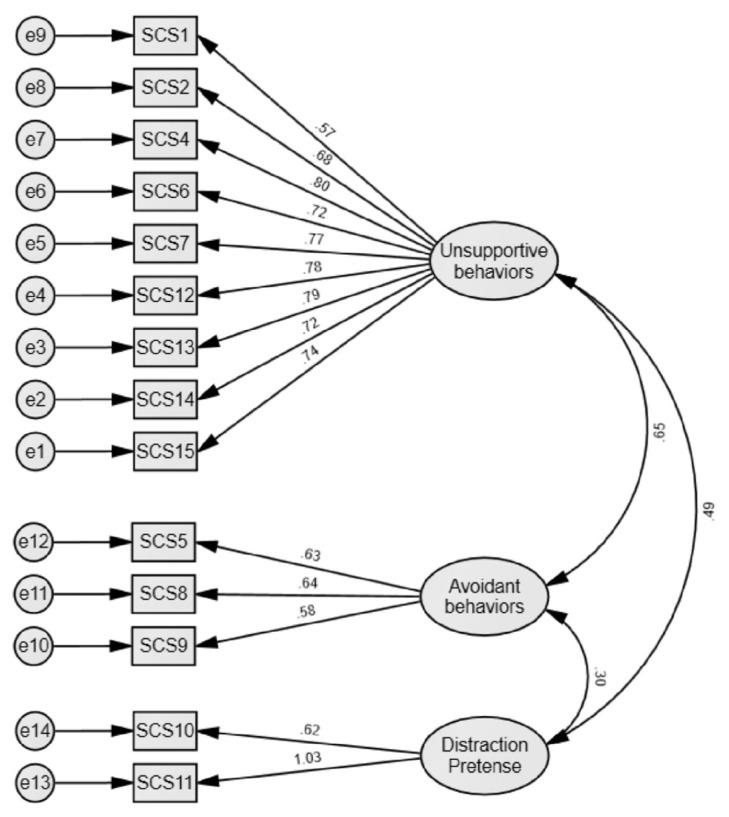
Measurement model of CFA of the 14 items of C-SCS Unsupportive behaviours = unsupportive behaviours factor; Avoidant behaviours = avoidant behaviours factor; Distraction Pretence = Suggestions for distraction and pretence factors; SCS1~14 = items 1~14; e1~14 = latent variables; The arrows represent correlations; Solid lines denote significant correlations; Numbers next to the latent factors or items represent correlation coefficients

CFA with the maximum likelihood method was conducted using the following three factors: The results showed a good overall fit of the model (SRMR = 0.07, RMSEA = 0.10, CFI = 0.88, GFI = 0.83, *P* < 0.001). Therefore, a three-factor model solution was found to be the most interpretable. All factor loadings were statistically significant, with most items showing values greater than 0.50, except for item 3 (i.e., Avoided you), which loaded on the unsupportive behaviours factor. Following this, a CFA was rerun after removing item 3 due to its low factor loading, and the model remains satisfactory with the three-factor model; all factors were statistically significantly loaded with values greater than 0.50, as shown in Figure 1.

## Discussion

The present study evaluated the psychometric properties of the C-SCS administered to a sample of literate postpartum mothers in Malaysia. While most studies have treated the SCS as a unidimensional measure (5, 11–13), a study by Koutrouli et al. (14) proposed a three-factor structure of the SCS, with subscales namely unsupportive behaviours, avoidant behaviours, and distraction and pretence suggestions. The EFA findings supported the three-factor structure of the SCS, with factor loadings ranging from 0.53 to 0.88, and no items were omitted. Nevertheless, the items demonstrated differences in their factor loadings, implying that the SCS structure varies slightly across different cultural backgrounds and contexts. Further research is needed to verify factor loadings in different cultural contexts.

Compared with the study by Koutrouli et al. (14), which was conducted on 202 women with breast cancer, items 1 (Changed the subject), 12 (Did not want to hear about your feelings), 13 (Felt uncomfortable and made you keep feelings to yourself), and 14 (Felt upset and made you keep feelings to yourself), which were initially loaded on the second factor, namely, avoidant behaviours, were now loaded on the first factor, which is unsupportive behaviours, leaving only three items loading on the factor of avoidant behaviours. Additionally, items 5 (Hid their feelings) and 8 (Complained about their own problems), which initially loaded on the first factor, were loaded on the avoidant behaviours factor, leaving only 10 items loading on the unsupportive behaviours factor. Furthermore, item 9 (Acted cheerful), which was initially loaded on the distraction and pretence suggestions factor, was loaded on the avoidant behaviours factor.

Interestingly, the findings showed that two out of three items loaded on the unsupportive behaviours factor. CFA was performed to confirm whether the three-factor structure fits the data well. The findings showed that although a three-factor model fits the data well, the analysis indicated that item 3 (Avoided you) loaded poorly on the unsupportive behaviours factor in CFA. Therefore, another round of CFA was performed with item 3 omitted, and the item loadings were satisfactory, suggesting that a 14- item model might be a better fit for postpartum mothers. The variation in factor loadings, compared with Koutrouli et al. (14) is likely attributed to the unique context of postpartum mothers in which the scale was applied. Additionally, sociocultural differences could influence these variations, affecting participants’ comprehension of the scale.

Moreover, the acceptable internal consistency provided support for construct validity, suggesting that the C-SCS is a valid measure to assess postpartum mothers’ experiences of social constraints. Furthermore, the positive correlation between the C-SCS and postpartum depression (i.e., EPDS) supported the validity of the C-SCS. Thus, postpartum women facing social constraints may experience greater depressive symptoms. In particular, previous analyses have supported the relationship between social constraint and psychological distress. According to Adams et al. (45), social constraint are a unique construct associated with greater psychological distress. Conversely, discriminant validity was established given the absence of correlations between the C-SCS and the PSQ.

This study provides a reference for postpartum mothers using the C-SCS. Overall, this study enriches the existing literature by validating the C-SCS as a useful measure for assessing the experiences of postpartum mothers with social constraints. Additionally, it provides evidence that the SCS is multidimensional. Interventions aimed at reducing social constraints could enhance psychosocial care for postpartum mothers experiencing distress.

This study has several limitations. First, certain reliability (e.g., test–retest reliability) was not assessed, which should be included in future research. The use of self-report measures in this study may introduce social desirability or short-term recall bias. Future studies should consider using other sampling methods, such as informant reports, to obtain additional data and perspectives from other sources (e.g., friends, partners, and family). Additionally, the sampling was limited to married Malaysian postpartum mothers who gave birth to healthy babies within their first postpartum year, indicating that caution is needed when generalising the findings to other populations. Examining the psychometric properties of the C-SCS in postpartum mothers from various countries, with different marital statuses, babies’ conditions, and at different postpartum periods, is important to improve its applicability to wider populations.

## Conclusion

In conclusion, the study indicated that the C-SCS is a reliable instrument that can be used to measure the experiences of social constraints among literate postpartum mothers in Malaysia during their first postpartum year. The findings supported the three-factor model, including unsupportive behaviours, avoidant behaviours, and suggestions for distraction and pretence, as the best fit for literate postpartum mothers in Malaysia. These findings suggest that the C-SCS could be useful for assessing social constraints perceived by mothers in healthcare settings.

## Figures and Tables

**Table 1:** Socio-demographic characteristics of participants

Variables	*n*	%	Mean	SD
**Age (years)**			29.82	4.28

19.0 to 24.5	13	10.0		
24.6 to 29.5	53	40.8		
29.6 to 34.5	44	33.8		
34.6 to 40.0	20	15.4		

**Religion**				

Buddhism	115	88.5		
Christianity	10	7.7		
Atheist/free thinker	5	3.8		

**Years of marriage**			3.87	3.00

≤ 1	4	3.1		
1 to 5	94	72.3		
6 to 10	26	20.0		
≥ 10	6	4.6		

**Education attainment**				

Primary school	3	2.3		
SPM/O levels or equivalent	40	30.8		
STPM/A levels/diploma or equivalent	25	19.2		
Bachelor’s degree or equivalent	52	40.0		
Postgraduate degree (Master’s/PhD)	10	7.7		

**Monthly household income**				

≤ RM 4,850	36	27.7		
RM 4,850 to RM 10,959	69	53.1		
≥ RM 10,959	25	19.2		

**Employment status**				

Employed	94	72.4		
Unemployed	36	27.6		

**Living arrangement**				

Living with only partner or children or both (nuclear family)	56	43.1		
Living with family members other than partner or children (extended family)	74	56.9		

**Parity**				

Primiparous	74	56.9		
Multiparous	56	43.1		

**Mode of delivery**				

Natural unassisted vaginal birth	72	55.4		
Assisted vaginal birth	17	13.1		
Planned caesarean section	17	13.1		
Emergency caesarean section	24	18.5		

Total	130	100.0		

Currency conversion rate is USD 1 = RM 4.18, as of 7 November 2025; Rate obtained from Xe

**Table 2:** Promax rotated factor matrix (principal component method) of C-SCS items (*n* = 130)

Item		Factor 1 Unsupportive behaviours	Factor 2 Avoidant behaviours	Factor 3 Suggestions for distraction and pretence
SCS1	Changed the subject	0.530		
SCS2	Did not understand your situation	0.690		
SCS3	Avoided you	0.586		
SCS4	Minimised your feelings	0.884		
SCS5	Hid their feelings		0.686	
SCS6	Acted uncomfortable	0.709		
SCS7	Trivialised your feelings	0.813		
SCS8	Complained their own problems		0.671	
SCS9	Acted cheerful		0.787	
SCS10	Told you not to worry so much			0.860
SCS11	Told you not to think about feelings			0.806
SCS12	Did not want to hear about your feelings	0.829		
SCS13	Felt uncomfortable and made you keep feelings to yourself	0.786		
SCS14	Felt upset and make you keep feelings to yourself	0.689		
SCS15	Did not show concern	0.680		

Only factor loadings ≥ 0.40 are shown
